# Association of cardiovascular risk using non-linear heart rate variability measures with the framingham risk score in a rural population

**DOI:** 10.3389/fphys.2013.00186

**Published:** 2013-07-26

**Authors:** Herbert F. Jelinek, Hasan Md Imam, Hayder Al-Aubaidy, Ahsan H. Khandoker

**Affiliations:** ^1^Department of Biomedical Engineering, Khalifa University of Science, Technology and ResearchAbu Dhabi, UAE; ^2^Australian School of Advanced Medicine, Macquarie UniversitySydney, NSW, Australia; ^3^Centre for Research in Complex Systems and the School of Community HealthAlbury, NSW, Australia; ^4^Department of Electrical and Electronic Engineering, University of MelbourneMelbourne, VIC, Australia

**Keywords:** cardiovascular risk factor, non-linear hear rate variability analysis, tone-entropy, complex correlation meausres, Framingham risk factor, Poincare plot

## Abstract

Cardiovascular risk can be calculated using the Framingham cardiovascular disease (CVD) risk score and provides a risk stratification from mild to very high CVD risk percentage over 10 years. This equation represents a complex interaction between age, gender, cholesterol status, blood pressure, diabetes status, and smoking. Heart rate variability (HRV) is a measure of how the autonomic nervous system (ANS) modulates the heart rate. HRV measures are sensitive to age, gender, disease status such as diabetes and hypertension and processes leading to atherosclerosis. We investigated whether HRV measures are a suitable, simple, noninvasive alternative to differentiate between the four main Framingham associated CVD risk categories. In this study we applied the tone-entropy (T-E) algorithm and complex correlation measure (CCM) for analysis of HRV obtained from 20 min. ECG recordings and correlated the HRV score with the stratification results using the Framingham risk equation. Both entropy and CCM had significant analysis of variance (ANOVA) results [*F*_(172, 3)_ = 9.51; <0.0001]. Bonferroni *post hoc* analysis indicated a significant difference between mild, high and very high cardiac risk groups applying tone-entropy (*p* < 0.01). CCM detected a difference in temporal dynamics of the RR intervals between the mild and very high CVD risk groups (*p* < 0.01). Our results indicate a good agreement between the T-E and CCM algorithm and the Framingham CVD risk score, suggesting that this algorithm may be of use for initial screening of cardiovascular risk as it is noninvasive, economical and easy to use in clinical practice.

## Introduction

Identification of the risk of a cardiovascular disease (CVD) is an important attribute of preventative health care.

### The framingham risk equation and cardiovascular risk

Multifactorial factors contribute to the increased risk of CVD. Previous health practice guidelines recommend that patients be treated with respect to their underlying coronary heart disease risk (Sheridan et al., 2003). Therefore accurate estimates are required to provide information on treatment strategies and timing of commencement of treatment. The Framingham risk equation has been validated in general populations. (D'Agostino et al., 2001) The Framingham risk equation has also been modified by various countries such as the Australian and New Zealand Cardiovascular Society (Jackson, 2000) as well as alternative risk equations proposed including the Coronary Risk Evaluation (SCORE) for fatal coronary heart disease or CVD and the Diabetes Epidemiology: Collaborative Analysis of Diagnostic Criteria in Europe (DECODE), which incorporates glucose tolerance status and fasting plasma glucose as well. (Conroy et al., 2003), (Balkau et al., 2004) History of CVD, physical inactivity, obesity, and left ventricular hypertrophy diagnosed by echocardiography are not included in current models of cardiovascular risk assessment (Jackson, 2000) as their individual predictive value is unclear, although obesity and left ventricular hypertrophy (LVH) are associated with increased cardiac pathology. There are suggestions that additional clinical measures may be of benefit such as biomarkers including C-reactive protein or D-dimer. (Charo et al., 1998; De Lemos, 2006; Nwose et al., 2007) However, these measures are invasive as they require blood samples to be sent to analytical laboratories and time consuming.

### The framingham score and autonomic nervous system

Assessment of the autonomic nervous system (ANS) function can provide information on the risk and presence of CVD. As such blood pressure, one component of the Framingham risk score (FRS) is a function of heart rate and peripheral vascular resistance. In turn heart rate is a function of metabolism, which is also in part regulated by the ANS. Peripheral vascular resistance is equally regulated by the ANS, albeit only by the sympathetic branch. Increased cholesterol levels combined with oxidative stress due to for instance, increased free radicals leads to pathophysiological changes in the vascular endothelial cells and atherosclerosis, which also affects blood pressure. However increased blood pressure can also lead to atherosclerosis (Alexander, 1995, p. 9301). The metabolic changes due to oxidative stress are particularly a factor in diabetes disease progression, where an increase in reactive oxygen species activity associated with an increase in blood glucose levels leads to not only lipid peroxidation and atherosclerosis but also to damage to the kidneys, eye, gastrointestinal tract and the vascular system. Similar pathological changes to the peripheral circulation are due to smoking. The final parameter contained in the Framingham equation is age. Aging also affects heart rate variability (HRV), with increasing age associated with a decrease in HRV. (Pikkujämsä et al., 1999) These factors are the standard clinical parameters incorporated into the Framingham risk equation. Therefore we hypothesized that parameters that indicate characteristics of ANS function such as ANS influence on heart rhythm may be good indicators for CVD risk.

The Framingham-based cardiovascular risk categories can be determined on line at http://www.mdcalc.com/framingham-coronary-heart-disease-risk-score-si-units/ and other websites. The Framingham risk equation uses data on gender, age, cholesterol, blood pressure, diabetes status and smoking to determine the 10 year risk of belonging to one of four risk categories: mild, moderate, high and very high (Wilson et al., 1998).

### HRV indices and framingham risk score

HRV is an important physiological factor that is regulated by the sinoatrial node located in the right atrium of the heart and connected to the remainder of the heart by an elaborate network of cardiac cells that conduct the electrical impulse throughout the heart muscle. The endocrine system via a diverse set of hormones and the ANS also modulate HRV (Naschitz et al., 2005). HRV is also influenced by the same factors as incorporated in the Framingham risk equation including age, gender, cholesterol, blood pressure, diabetes status and smoking.

Standards of measurement and interpretation of HRV have been recommended by the Task Force of the European Society of Cardiology and the North American Society of Pacing and Electrophysiology (Tfesc/Naspe, 1996). However the majority of studies investigating cardiovascular risk and HRV have used clinical data such as from the Framingham cohort to assess correlations between parameters measured as part of the FRS and HRV. Thus in the Framingham Heart Study, frequency and time domain analysis of HRV were found to be associated with a higher CVD mortality. (Tsuji et al., 1996) Studies by our laboratory and others have investigated the correlation between HRV and hypertension, cholesterol, gender, age, hyperglycemia and diabetes, smoking, body mass index, oxidative stress and heart disease. (Da Silva et al., 2004; Lampert et al., 2008; McLachlan et al., 2010; Tacoy et al., 2010; Thayer et al., 2010; Johnson et al., 2011; Khandoker et al., 2011; Kotecha et al., 2011; Fakhrzadeh et al., 2012; Matthews et al., 2012; Thiyagarajan et al., 2013) However, these studies did not stratify the cohorts according to the FRS but rather evaluated the magnitude of the HRV associated with presence or absence of the pathology being investigated. Hillebrand et al. (2013) recently reported that HRV is a useful marker for incidence of CVD in a population without any signs of CVD based on lowered time domain HRV values (Hillebrand et al., 2013).

Our search of the literature found one previous paper that investigated the correlation between HRV and FRS in a group of healthy adults (Yoo et al., 2011). The results of the study was based on dichotomizing the FRS into two groups, one with a CVD risk lower than 10% and the other a CVD risk higher than 10%. The results indicated that both time and frequency domain analyses were useful parameters for differentiating between high and low CVD risk.

Time and frequency domain analyses are sensitive to the length and non-stationarity inherent in the R-R series obtained from the ECG recordings. Several HRV analysis methods have been proposed in the last 10 years, which address some of the shortcomings associated with time and frequency domain analysis. The tone-entropy (T-E) algorithm is a method that is robust against data length, non-stationarity of the signal and also against respiratory influence and has been validated as a tool for analyzing HRV. The T-E algorithm has also been shown to correlate with experimentally induced HRV changes by either blocking the parasympathetic component using atropine or increasing the sympathetic influence on heart rate by head-up tilt (Oida et al., 1999). A clinical study analyzing HRV in a cohort of people with diabetes and cardiac autonomic neuropathy indicated a good correlation between cardiac autonomic progression and T-E as suggested by the work of Oida and colleagues (Khandoker et al., 2010). T-E is a combination of tone, which indicates the sympathovagal balance and entropy which indicates the overall activity of the ANS. Higher tone and lower entropy values indicate worsening ANS function and reduced HRV, which by its nature has a certain amount of variability and activity level (Khandoker et al., 2010).

Current HRV parameters used in HRV analysis are essentially static measures of a time signal. To obtain a better understanding of the relationship between autonomic control of the heart rate and the correlation to CVD risk a more dynamic measure of heart rate is required (Peng et al., 1995). The Poincaré Plot is a method proposed and utilized by Tulppo et al. (1996) for analysis of heart rate signals. The Poincaré Plot allows determining linear components of the inter-beat variability associated with short and long term correlations of the signal (Tulppo et al., 1998; Karmakar et al., 2009a). An extension of the Poincaré Plot to determine and measure the temporal dynamics over the recording interval was proposed by Karmakar et al. (2011). The complex correlation method (CCM) relies on computing the point-to-point variation of the signal with increasing lag (beat intervals) rather than the global description the Poincare plot provides based on *n*, and *n* + 1 beats.

This study investigated whether T-E and CCM are able to differentiate between ECG recordings obtained from four groups of patients categorized according to their FRS into mild, moderate, high and very CVD risk.

## Methods

### Framingham risk assessment and ECG signals

Data for the study was obtained from the Diabetes Screening Complications Initiative (DiScRi) clinic at Charles Sturt University. The study was approved by the Charles Sturt University Human Ethics Committee and undertaken between 2004 and 2008. Patient data was only included if it was complete for demographic as well as experimental variables. Under these criteria, 319 records and ECG traces were available. The Framingham cardiac risk score was determined using the protocol outlined by Jackson (2000) and determined automatically from data entered into the DiScRi ACCESS database (Pecoul and Jelinek, 2008). The variables used for determination of the cardiac risk score were age, sex, blood pressure, cholesterol [total and high density lipoprotein (HDL)], and smoking with diabetes status being a categorical variable. Cardiac risk as defined by the Framingham risk score is divided into very high (>20%), high (15–20%), moderate (10–15%) and mild (less than 10%) in accordance with the Australian and New Zealand Guidelines. The CVD risk score for each patient was used to categorize them into one of the four groups.

ECG signals were recorded and edited using the MLS310 HRV module (version 1.0, ADInstruments, Australia) included in the Chart software package. High frequency noise was removed with a 45 Hz low-pass filter and a 0.5 Hz high pass filter adjusted for wandering baseline. Ectopic beats were selected visually and deleted manually. Linear interpolation was used to replace ectopic beats that occur immediately before and after the ectopic interval. Intervals between successive R waves of the QRS complex (i.e., R-R intervals in seconds) were calculated using the algorithm developed by Pan and Tompkins.(Tompkins, 1993) The HRV analysis described in the following sections was performed on 1000 RR intervals.

### Time and frequency domain analysis

We quantified several time domain HRV parameters: mean RR, standard deviation of normal RR data (SDNN) and the square root of the mean squared difference of the successive RR data (RMSSD). Spectral analysis was performed on linearly resampled (1 Hz) time series using Welch's method (Welch, 1967). The 256-point fast Fourier transform was repeatedly computed with 50% overlap between adjacent segments. Then the spectral power of each segment was computed and averaged. Hanning window was applied to avoid spectral leakage. Subsequently, spectral powers in the low frequency (LF) band (0.04–0.15 Hz) and high frequency (HF) band (0.15–0.40 Hz) were obtained by integration (TFESC/NASPE). The normalized LF and HF powers were calculated by LF/(Total Power–VLF) and HF/(Total Power–VLF) respectively as per Task Force recommendation. (Tfesc/Naspe, 1996) The power in the very lower frequency (VLF) band was set at ≤ 0.04 Hz.

### Tone-entropy determination

The methodology was described in detail in previous reports (Oida et al., 1999; Amano et al., 2005). In brief, acquired heart periods (RR intervals) are transformed into percentage index (*PI*) time series:

(1) PI(n)=[H(n)−H(n+1)]×100/H(n)

where *H(n*) is a heart period time series, and *n* a serial number of heart beats shown. *PI(n)* therefore represents the number of instances each specific heart rate interval (RR) occurs in the time series. The tone is defined as a first order moment (arithmetic average) of this *PI* time series as:

(2) $$\sum_{n}PI(n)/N(\text{non-dimensional})$$

where *N* is a total number of *PI* terms. The tone, balance between accelerations (*PI* > 0) and inhibitions (*PI* < 0) of the heart, represents the sympatho-vagal balance faithfully as appreciated in all the previous studies (2002). The entropy is defined on *PI* probability distribution by using Shannon's formula:

(3) $$-\sum_{n}p(i)\log_{2}p(i)\text{(bit)}$$

where [*p(i)*] is a probability that *PI(n)* has a value in the range, *i* < *PI* (*n*) < *i* + 1, *i* an integer. The entropy evaluates total acceleration–inhibition activities, or total heart period variations, in a familiar unit of bit. Entropy represents therefore the autonomic regulatory activity and tone the sympatho-vagal balance (Khandoker et al., 2010).

Tone as a feature has its origin in the investigations in the last century of Rosenbluth and Simeone (Rosenblueth and Simeone, 1984). These authors investigated autonomic control of heart rate as an antagonistic interactive operation between acceleration and inhibition. Entropy evaluates HRV almost the same way as conventional second-order moments, for example, as standard deviation and is based on the Shannon entropy.

### Complex correlation measure

CCM measures the point-to-point variation of the signal rather than gross description of the Poincaré plot. It is computed in a windowed manner, which embeds the temporal information of the signal. A moving window of three consecutive points from the Poincaré plot are considered and the temporal variation of the points are measured. If three points are aligned on a line then the value of the variation is zero, which represents the linear alignment of the points. If the Poincaré plot is composed of *N* points then the temporal variation of the plot, the CCM, is composed of all overlapping three point windows and can be calculated as:

(4) $$\text{CCM}(m)=\frac{1}{C_{n}(N-2)}\sum_{i=1}^{N-2}||A(i)||$$

where *m*, the lag, represents the number of consecutive points used from the Poincaré plot, *A(i)* represents area of the *i*-th triangle and *C*_*n*_ is the normalizing constant which is defined as, *C*_*n*_ = π^*^ SD1^*^ SD2, represents the area of the fitted ellipse over Poincaré plot at lag-m. The length of major and minor axis of the ellipse are 2SD1, 2SD2, where SD1, SD2 are the dispersion perpendicular to the line of identity (minor axis) and along the line of identity (major axis) respectively as proposed by Tulppo et al. (1996) The detail mathematical formulation of CCM is reported in our previous study Karmakar et al. (2009b).

### Statistical analysis

Results were expressed as means (±SD). One-way analysis of variance (ANOVA) and Bonferroni *post hoc* examination were carried out for comparisons among the four groups (mild, moderate, high, and very high) to evaluate whether statistically significant differences exist among the groups. In this study, the Lilliefors test was applied to test if the HRV features (tone, entropy, and CCM) comes from a distribution in the normal family. Results from the Lilliefors tests confirmed that HRV features' distributions were normal. Therefore, we decided to perform the statistical analysis with ANOVA. A value of *p* < 0.05 was considered significant for all examinations.

## Results

Three hundred and nineteen patients were recruited into the study with complete results from the DiScRi clinic including data required for determining the FRS. After exclusion of results due to noise in ECG signals or ectopic beats, 170 patients were included in the final analysis. Of these 85 participants were identified with mild (57%), 37 with moderate (18%), 25 with high (13%), and 23 (12%) cardiovascular risk. Demographic and cardiac risk factors according to the Framingham model are shown in Table 1 (Lloyd-Jones, 2010).

**Table 1:** **Study population framingham CVD risk parameters and demographics**.

	**Mild**	**Moderate**	**High**	**Very high**	***P*-values**
Number	85	37	25	23	
Gender, M(F)	25 (60)	16 (21)	16 (9)	14 (9)	
Age (years)	60.2 ± 12.4	63.1 ± 12.4	66.5 ± 11.4	66.1 ± 12.4	0.020
SBP(mmHg)	126.5 ± 16	132.6 ± 16.7	132.3 ± 17.7	141.3 ± 18.8	0.001
TC/HDL(mmol/L)	3.6 ± 1.1	4.2 ± 1.1	2.78 ± 1.1	3.5 ± 1.2	0.040
DM, yes(no)	22 (63)	15 (22)	12 (13)	16 (7)	
BMI	27.5 ± 6.3	28.2 ± 3.6	26.1 ± 4.9	27.7 ± 5.7	0.451
HbA1c(%)	6.1 ± 0.8	6.2 ± 0.6	6.2 ± 1	6.10 ± 0.5	0.100
HT, yes(no)	40 (45)	16 (21)	15 (10)	13 (10)	

Results are provided as mean ± SD; BMI, body mass index; HbA1c, glycosylated hemoglobin; TC/HDL, total cholesterol to high density lipoprotein cholesterol; SBP, systolic blood pressure; DM, diabetes mellitus; HT, hypertension.

Both entropy and CCM had significant ANOVA results [*F*_(172, 3)_ = 9.51; <0.0001]. Bonferroni *post hoc* analysis indicated a significant difference between mild, high and very high cardiac risk groups with the entropy feature (Table 2). CCM detected a difference in temporal dynamics of the RR intervals between the mild and very high CVD risk groups.

**Table 2:** **Heart rate variability results for CVD risk groups**.

**HRV features**	**Mild (85)**	**Moderate (37)**	**High (25)**	**Very high (23)**	***p***
Tone	−0.584 ± 0.783	−0.388 ± 0.586	−0.265 ± 0.515	−0.319 ± 0.349	0.070
Entropy	2.476 ± 0.630	2.176 ± 0.567	2.007 ± 0.699^*^	1.699 ± 0.530^**^	0.0001
CCM	0.526 ± 0.384	0.430 ± 0.347	0.314 ± 0.141	0.280 ± 0.180^**^	0.001
SDNN(ms)	50.33 ± 24.13	46.97 ± 18.32	39.68 ± 18.37	38.64 ± 13.38^**^	0.05
RMSSD(ms)	46.23 ± 28.08	43.04 ± 21.94	39.12 ± 23.21	38.41 ± 17.82	0.51
VLF(s^2^)	0.80 ± 0.81	0.72 ± 0.56	0.54 ± 0.59	0.44 ± 0.27	0.13
LF(nu)	0.60 ± 1.09	0.48 ± 0.55	0.41 ± 0.40	0.32 ± 0.41	0.34
HF(nu)	1.05 ± 1.65	0.90 ± 0.89	0.69 ± 0.77	0.65 ± 0.64	0.53
LF/HF	0.59 ± 0.30	0.59 ± 0.28	0.82 ± 0.63	0.63 ± 0.58	0.65

* p < 0.01 for Mild vs. High;

** p < 0.01 for Mild vs. Very high.

**Figure 1:**
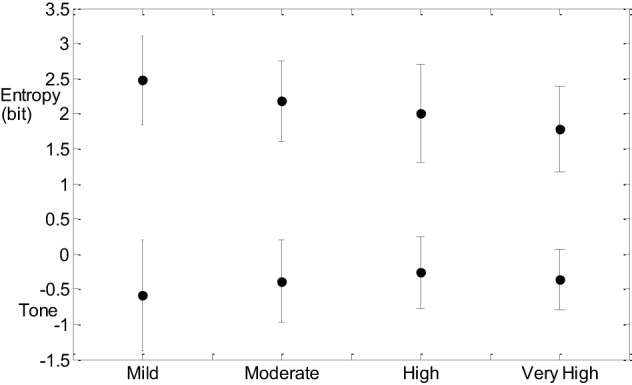
**Tone and entropy mean ± SD values for each of the four CVD risk groups**.

*Post hoc* analysis of SDNN for the CVD risk groups was also significant at 0.05 between mild and very high CVD risk. Figure 1 indicates that entropy steadily decreases with increased CVD risk and tone increases with increasing CVD risk (Figure 1).

**Figure 2:**
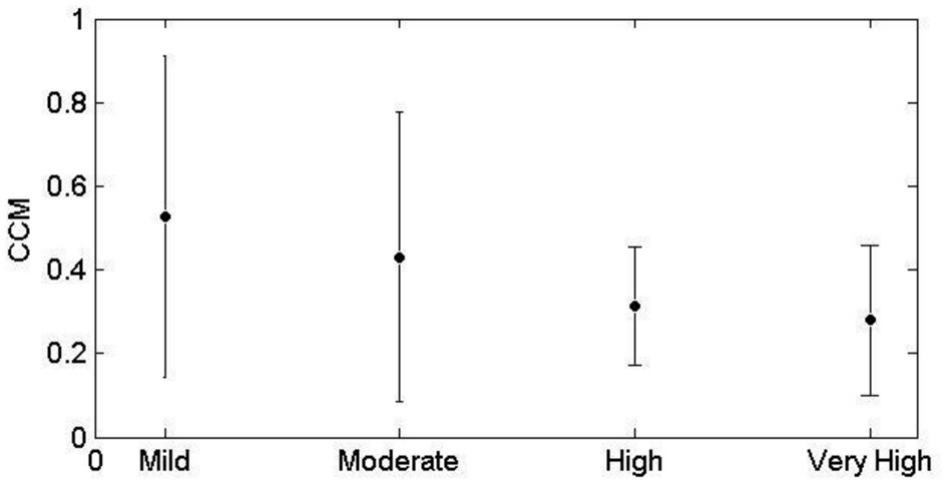
**CCM mean ± SD values for each of the four CVD risk groups**.

CCM similarly decreases with advance of CVD risk (Figure 2). The increase in tone indicates a reduction in sympathovagal balance and the decrease in CCM indicates that this reduction in sympathovagal balance may be due to a reduction in parasympathetic influence on HRV. Entropy decreases with CVD risk progression, indicating a loss of ANS activity.

**Figure 3:**
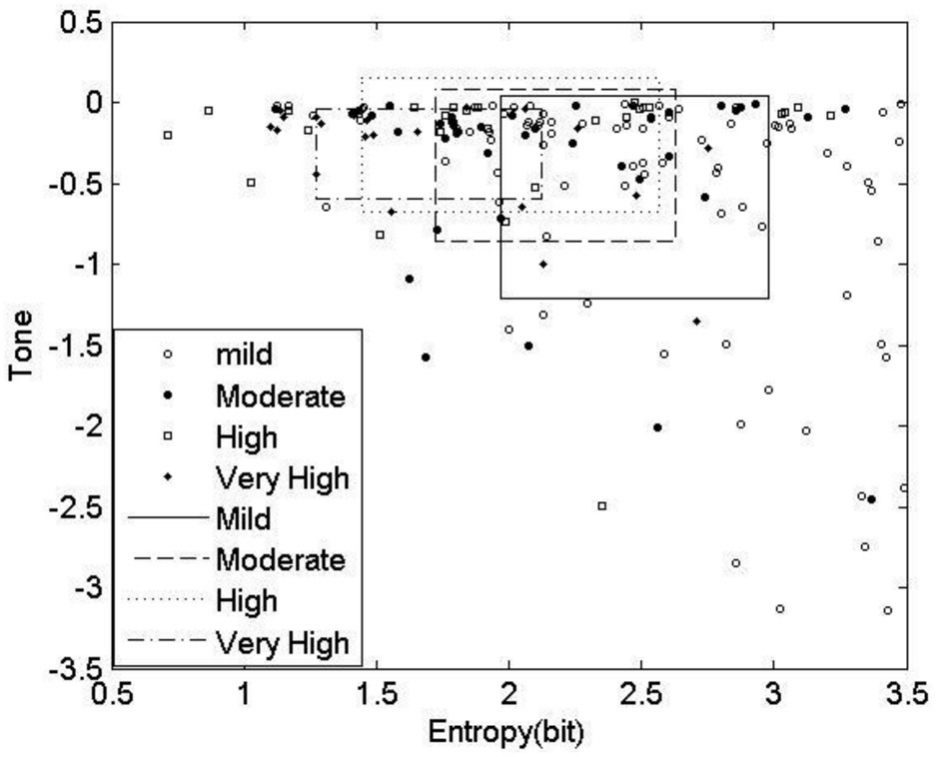
**Tone-Entropy plot of all subjects in four groups**.

The results of the T-E analysis were then transformed into 2D space shown in Figure 3. Rectangles show the mean and standard errors of Tone and Entropy values of each group. The symbols show the tone-entropy results for each participant with respect to CVD risk category. The important finding is that the distribution of results on the T-E plot roughly follow a curvilinear trend from top left to bottom right, showing as outlined in the text that there is an increase in tone and a decrease in entropy as one goes from the mild CVD risk group to the very high risk group (see Table 1).

**Figure 4:**
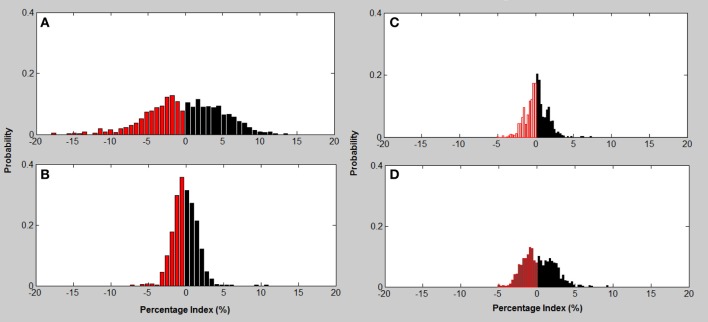
**Excitation—inhibition histograms associated with CVD risk category. (A)** mild; **(B)** moderate; **(C)** high; **(D)** very high risk; Excitation (black bars)—inhibition (red bars) histograms associated with CVD risk category.

Excitation-inhibition characterizes HRV and is the basis for calculating tone in the T-E algorithm. Figure 4 indicates the histograms based on the probability of excitations and inhibitions occurring in any heart rate recording. The histograms indicate that the relationship between excitations and inhibitions are clearly different between the four CVD risk groups (Figure 4).

The Mild CVD risk histogram has a wide spread of both acceleration and inhibitions in heart rate with inhibitions on the left being slightly more common. The moderate CVD risk group (B) indicates that there is a loss of both inhibitions and excitations with the remaining beat intervals occurring at a higher percentage. With high CVD risk (C) the variance (spread of the histogram) of inhibition and excitations are still reduced but in addition the percentage of the different beat lengths occurring is also reduced. The histogram for very high CVD risk (D) is characterized by a further reduction in variance or spread of the histogram and inhibitions and excitations all occur less often but with inhibitions dominating the heart rate variance.

## Discussion

CVD risk as determined by the FRS requires data for gender and age, diabetes status, blood pressure, cholesterol level as well as smoking status. From a clinical perspective determining the final Framingham risk score is based on analysis of blood samples, which is an invasive procedure and requires samples to be sent to a testing laboratory. Simpler methods that are non-invasive but are correlated with the FRS may provide an alternative where the FRS cannot be calculated or provide additional clinical information. Our study focused on whether the magnitude of CVD risk as determined by FRS is correlated with HRV measured by the T-E and CCM algorithms.

Current measures of the autonomic balance of heart rate regulation include time and frequency domain as well as nonlinear measures (Goldberger and West, 1987; Pincus, 1991; Alam et al., 2009; Karmakar et al., 2009a; Voss et al., 2009; Cysarz et al., 2011). Time and frequency domain measures provide a global characterization of the HRV over the recording interval, which can range between 2 min to 24 h (Dekker et al., 2000; Rennie et al., 2003; Kiviniemi et al., 2011). To obtain a better understanding of the optimal ANS control of the heart rate, more dynamic measures of heart rate such as nonlinear methods are required. Quantitative Poincaré plot analysis was used to assess the changes in CCM of HRV signals during parasympathetic blockade was discussed in our previous study (Karmakar et al., 2011). The lowest value of *CCM* has also been found during atropine infusion which reduced the parasympathetic activity and reduces instantaneous changes in HRV signal. On the other hand, it was also found to be increased with increase in parasympathetic activity during administration of low-dose scopolamine. Variability (increasing or decreasing) in the temporal structure of the Poincaré plot (measured as *CCM*) reflects the change in parasympathetic activity harmoniously. An advantage of nonlinear methods is that they are not sensitive to the non-stationarity of ECG signals (Peng et al., 1995). T-E has a number of advantages over the conventional methods of HRV analysis in that it is robust against non-stationarity and respiratory influence and not sensitive to differences in length of recordings. Similarly CCM can be applied to short recordings and provides temporal/dynamic information about HRV not obtained by other methods (Karmakar et al., 2011). This latter point makes tone, entropy and CCM especially useful for clinical investigation where short time recordings are more likely to be used. T-E provides also an alternative visualization of the functionality of the ANS with respect to CVD risk as shown in Figure 2 and CCM is a dynamic rather than a static measure of HRV. CCM therefore provides information on the magnitude of the beat-to-beat influence from a lag of one beat to ten beats, which has been shown to be important in HRV and is subject to parasympathetic influence (Lerma et al., 2003), necessitating a means of exploring this influence. CCM, rather than analyzing beat-to-beat variation in fact analyses the relationship between an initial beat and any beat downstream from *n* to *n* + 1. In this work we have used *n* = 3.

Our results show that mild risk of less than 10%, moderate (10–15%), high (15–20%) and very high risk (>20%), in accordance with the Australian and New Zealand Guidelines, is associated with decreasing entropy and CCM and an increasing tone. The lack of a significant result between mild and moderate may be in part due to the minor difference in CVD risk % between the current groups. Previous work by Yoo et al. (2011) divided the cohort into those with less or greater than 10% CVD risk and showed a significant relationship between the FRS and time and frequency domain parameters (Yoo et al., 2011). Our analysis using the four main CVD risk groups of the FRS showed only a significant result for SDNN, the global time domain measure. No frequency domain measures were significant. We propose that entropy and CCM are more suitable measures to identify CVD risk in terms of the FRS due to their robust nature against non-stationarity and measuring total activity and the dynamic nature of the heart rate interval changes over the time of the recording.

Analysis of the distribution of accelerations and inhibitions as shown in Figure 4 are related to the tone-entropy results. The mild CVD risk group has the widest histogram and the lowest peak, which indicates the highest entropy and lowest tone and therefore a higher HRV. Mild CVD risk has the most active sympatho-vagal modulation of heart rate and optimum sympatho-vagal balance. Narrower histograms as seen in Figure 4 with higher peaks indicate loss of ANS activity by a decrease in entropy and loss of sympatho-vagal balance indicated by the increase in tone. The trend of increasing tone and decreasing entropy continues as CVD risk increases. Moderate, high and very high CVD risk histograms are narrower with higher tone values and therefore a lower sympatho-vagal balance. This is especially prominent in the moderate CVD risk group. T-E analysis thus indicates subtle physiological changes in heart rate modulation not previously identified. These results correlate with previous physiological and pharmacological experiments (see Figures 2, 8; Oida et al., 1997).

The results for T-E and CCM analysis correlate with the CVD risk as determined by the FRS. HRV changes in response to various factors including ANS activity. In turn ANS activity is dependent on age, smoking, diabetes and blood pressure, all being variables of the Framingham CVD risk equation. TE and CCM are able to differentiate the mild to high and very high CVD risk groups as defined by the Framingham CVD risk score. As such HRV parameters may be seen as a single measure that is non-invasive and provides an alternative for identifying CVD risk. However, we are not suggesting a new or better classification of risk compared to the FRS but rather that T-E and CCM correlate well with the accepted Framingham CVD risk score and the associated categories and provide a better stratification compared to the time and frequency domain parameters as can be seen in Table 2. In essence we are proposing that the entropy and CCM measure are able to stratify CVD risk groups as defined by FRS to mild, high and very high CVR risk groups. This has the advantage that a noninvasive, easy to perform method can be used in the first instance to identify individuals with increased risk of CVD. This becomes important when blood samples for instance cannot be taken for ethical, ethnic or other reasons. In addition HRV has been shown to be a sensitive marker for sudden cardiac death and also for inflammation as an atherosclerosis process as well as risk of arrhythmic events.

A future study of ours is to determine tone, entropy and CCM in a larger, younger cohort as most people below 40 are unlikely to have a very high risk of CVD and therefore may be missed in traditional CVD risk assessment despite other factors included in the Framingham risk equation being abnormal. In addition we are investigating the individual influence of the Framingham CVD risk factors on HRV either separately or in combination. This will provide a basis for reviewing younger patients with raised blood pressure and raised cholesterol levels but in a low CVD risk category due to age.

### Conflict of interest statement

The authors declare that the research was conducted in the absence of any commercial or financial relationships that could be construed as a potential conflict of interest.

## References

[B1] Alam I. Lewis M. J. Morgan J. Baxter J. (2009). Linear and nonlinear characteristics of heart rate time series in obesity and during weight-reduction surgery. Physiol. Meas. 30, 541–557 10.1088/0967-3334/30/7/002 19458410

[B1a] Alexander R. W. (1995). Hypertension and the pathogenesis of atherosclerosis: oxidative stress and the mediation of arterial inflammatory response: a new perspective. Hypertension 25, 155–161 10.1161/01.hyp.25.2.155 7843763

[B2] Amano M. Oida E. Moritani T. (2005). Age-associated alteration of sympatho-vagal balance in a female population assessed through the tone-entropy analysis. Eur. J. Appl. Physiol. 94, 602–610 10.1007/s00421-005-1364-x 15942770

[B4] Balkau B. Hu G. Qiao Q. Tuomilehto J. Borch-Johnson K. Pyorala K. (2004). DECODE Study Group: prediction of the risk of cardiovascular mortality using a score that includes glucose as a risk factor: the DECODE Study. Diabetologia 47, 2118–2128 10.1007/s00125-004-1574-5 15662552

[B5] Charo S. Gokce N. Vita J. (1998). Endothelial dysfunction and coronary risk reduction. J. Cardiopulm. Rehabil. 18, 60–67 10.1097/00008483-199801000-00008 9494884

[B6] Conroy R. M. Pyorala K. Fitzgerald A. P. Sans S. Menotti A. De Backer G. (2003). Estimation of ten year risk of fatal cardiovascular disease in Europe: the SCORE project. Eur. Heart J. 24, 987–1003 10.1016/S0195-668X(03)00114-3 12788299

[B7] Cysarz D. Linhard M. Edelhäuser F. Längler A. Van Leeuwen P. Henze G. (2011). Unexpected course of nonlinear cardiac interbeat interval dynamics during childhood and adolescence. PLoS ONE 6:e19400 10.1371/journal.pone.0019400 21625487 PMC3098842

[B8] D'Agostino R. B. Grundy S. M. Sullivan L. M. Wilson P. (2001). Validation of the Framingham coronary heart disease prediction scores. J. Am. Med. Assoc. 286, 180–187 11448281 10.1001/jama.286.2.180

[B9] Da Silva A. M. J. Moreira G. Daher M. T. (2004). Analysis of heart rate variability in hypertensive patients before and after treatment with angiotensin II converting enzyme inhibitors. Arq. Bras. Cardiol. 83, 169–172 15322659

[B10] De Lemos J. A. (2006). The latest and greatest new biomarkers: which ones should we measure for risk prediction in our practice? Arch. Intern. Med. 166, 2428–2430 10.1001/archinte.166.22.2428 17159006

[B11] Dekker J. M. Crow R. S. Folsom A. R. Hannan P. J. Liao D. Swenne C. A. (2000). Low heart rate variability in a 2-minute rhythm strip predicts risk of coronary heart disease and mortality from several causes: the aric study. Circulation 102, 1239–1244 10.1161/01.CIR.102.11.1239 10982537

[B12] Fakhrzadeh H. Yamini-Sharif A. Sharifi F. Tajalizadekhoob Y. Mirarefin M. Mohammadzadeh M. (2012). Cardiac autonomic neuropathy measured by heart rate variability and markers of subclinical atherosclerosis in early type 2 diabetes. ISRN Endocrinol. 2012, 7 10.5402/2012/168264 23259073 PMC3521488

[B13] Goldberger A. L. West J. B. (1987). Application of nonlinear dynamics to clinical cardiology, in Persectives in Biological Dynamics and Theoretical Medicine, eds Koslow S. Mandell A. Shlesinger M. (New York, NY: The New York Academy of Sciences), 195–213 10.1111/j.1749-6632.1987.tb48733.x 3477116

[B14] Hillebrand S. Gast K. B. De Mutsert R. Swenne C. A. Jukema J. W. Middeldorp S. (2013). Heart rate variability and first cardiovascular event in populations without known cardiovascular disease: meta-analysis and dose–response meta-regression. Europace 15, 742–749 10.1093/europace/eus341 23370966

[B15] Jackson R. (2000). Updated New Zealand cardiovascular disease risk-benefit prediction guide. BMJ 320, 709–710 10.1136/bmj.320.7236.709 10710588 PMC1117717

[B16] Johnson M. S. Demarco V. G. Heesch C. M. Whaley-Connell A. T. Schneider R. I. Rehmer N. T. (2011). Sex differences in baroreflex sensitivity, heart rate variability, and end organ damage in the TGR(mRen2)27 rat. Am. J. Physiol. Heart Circ. Physiol. 301, H1540–H1550 10.1152/ajpheart.00593.2011 21821781 PMC3197369

[B17] Karmakar C. Khandoker A. Voss A. Palaniswami M. (2011). Sensitivity of temporal heart rate variability in Poincare plot to changes in parasympathetic nervous system activity. Biomed. Eng. Online 10, 17 10.1186/1475-925X-10-17 21366929 PMC3061954

[B18] Karmakar C. K. Khandoker A. Gubbi J. Palaniswami M. (2009a). Complex correlation measure: a novel desciptor for Poincaré plot. Biomed. Eng. Online 8, Available online at: http://www.biomedical-engineering-online.com/content/8/1/17 10.1186/1475-925X-8-17 19674482 PMC2743693

[B19] Karmakar C. K. Khandoker A. H. Gubbi J. Palaniswami M. (2009b). Novel feature for quantifying temporal variability of Poincaré Plot: a case study Comput. Cardiol. 36, 53–56

[B20] Khandoker A. H. Jelinek H. F. Moritani T. Palaniswami M. (2010). Association of cardiac autonomic neuropathy with alteration of sympatho-vagal balance through heart rate variability analysis Med. Eng. Phys. 32, 161–167 10.1016/j.medengphy.2009.11.005 20004128

[B21] Khandoker A. H. Weiss D. N. Skinner J. E. Anchin J. M. Imam H. M. Jelinek H. F. (2011). PD2i heart rate complexity measure can detect cardiac autonomic neuropathy: an alternative test to Ewing battery, in Computing in Cardiology, Vol. 38 (China: IEEE Press). ISBN: 978-1-4244-4122-8/11

[B22] Kiviniemi A. M. Hautala A. J. Karjalainen J. Piira O.-P. Catai A. M. Mäkikallio T. H. (2011). Heart rate dynamics after exercise in cardiac patients with and without type 2 diabetes. Front. Physiol. 2:57 10.3389/fphys.2011.00057 21922009 PMC3166711

[B23] Kotecha D. New G. Flather M. D. Eccleston D. Pepper J. Krum H. (2011). Five-minute heart rate variability can predict obstructive angiographic coronary disease. Heart 98, 395–401 10.1136/heartjnl-2011-300033 22121069

[B24] Lampert R. Bremner J. D. Su S. Miller A. Lee F. Cheema F. (2008). Decreased heart rate variability is associated with higher levels of inflammation in middle-aged men. Am. Heart J. 156, 759 e751–759 e757 18926158 10.1016/j.ahj.2008.07.009 PMC2587932

[B25] Lerma C. Infante O. Perez-Grovas H. Jose M. V. (2003). Poincare plot indexes of heart rate variability capture dynamic adaptations after haemodialysis in chronic renal failure patients. Clin. Physiol. Funct. Imaging 23, 72–80 10.1046/j.1475-097X.2003.00466.x 12641600

[B26] Lloyd-Jones D. M. (2010). Cardiovascular risk prediction. Circulation 121, 1768–1777 10.1161/CIRCULATIONAHA.109.849166 20404268

[B27] Matthews S. Jelinek H. F. Vafaeiafraz S. McLachlan C. S. (2012). Heart rate stability and decreased parasympathetic heart rate variability in healthy young adults during perceived stress. Int. J. Cardiol. 156, 337–338 10.1016/j.ijcard.2012.02.004 22370369

[B28] McLachlan C. S. Ocsan R. Spence I. Hambly B. Matthews S. Wang L. (2010). HRV indices in association with physical activity and resting heart rate in bradycardia. BUMC Proc. 23, 368–370 10.1080/08998280.2010.11928655 PMC2943451 20944759

[B29] Naschitz J. E. Rozenbaum M. Fields M. Isseroff H. Enis S. Babich J. P. (2005). Search for disease-specific cardiovascular reactivity patterns: developing the methodology. Clin. Sci. 108, 37–46 10.1042/CS20040092 15330754

[B30] Nwose E. U. Richards R. S. Jelinek H. F. Kerr P. G. (2007). D-dimer levels reflect progression of diabetes mellitus and likelihood of cardiovascular complications. Pathology 39, 252–257 10.1080/00313020701230658 17454757

[B31] Oida E. Kannagi T. Moritani T. Yamori Y. (1999). Aging alteration of cardiac vagosympathetic balance assessed through the tone-entropy analysis. J. Gerontol. 54A, M219–M224 10362003 10.1093/gerona/54.5.m219

[B32] Oida E. Moritani T. Yamori Y. (1997). Tone-entropy analysis on cardiac recovery after dynamic exercise. J. Appl. Physiol. 82, 1794–1801 9173943 10.1152/jappl.1997.82.6.1794

[B33] Pecoul E. Jelinek H. F. (2008). A comprehensive electronic patient record for global risk assessment in a rural community, in BIOSTEC 2008, International Conference on Health Informatics, (Funchal, Madeira: IEE Press), 13–18

[B34] Peng C. K. Havlin S. Stanley H. E. Goldberger A. L. (1995). Quantification of scaling exponents and cross over phenomena in nonstationary heartbeat time series analysis. CHAOS 5, 82–87 10.1063/1.166141 11538314

[B35] Pikkujämsä S. M. Mäkikallio T. H. Sourander L. B. Räihä I. J. Puukka P. Skyttä J. (1999). Cardiac interbeat interval dynamics from childhood to senescence: comparison of conventional and new measures based on fractals and chaos theory. Circulation 100, 393–399 10.1161/01.CIR.100.4.393 10421600

[B36] Pincus S. (1991). Approximate entropy as a measure of system complexity. Proc. Natl. Acad. Sci. U.S.A. 88, 2297–2301 10.1073/pnas.88.6.2297 11607165 PMC51218

[B37] Rennie K. L. Hemingway H. Kumari M. Brunner E. Malik M. Marmot M. (2003). Effects of moderate and vigorous physical activity on heart rate variability in a British Study of civil servants. Am. J. Epidemiol. 158, 135–143 10.1093/aje/kwg120 12851226

[B38] Rosenblueth A. Simeone A. (1984). The interrelations of vagal and accelerator effects on the cardiac rate. Am. J. Physiol. 110, 42–55

[B39] Sheridan S. Pignone M. Mulrow C. (2003). Framingham-based tools to calculate the global risk of coronary heart disease. a systematic review of tools for clinicians. J. Gen. Int. Med. 18, 1039–1052 10.1111/j.1525-1497.2003.30107.x 14687264 PMC1494957

[B40] Tacoy G. Acikgoz K. Kocaman S. A. Ozdemir M. Cengel A. (2010). Is there a relationship between obesity, heart rate variability and inflammatory parameters in heart failure? J. Cardiovasc. Med. 11, 118–124 10.2459/JCM.0b013e328332e730 19809354

[B41] Tfesc/Naspe, (1996). Heart rate variability. Standards of measurement, physiological interpretation, and clinical use. Task Force of the European Society of Cardiology and the North American Society of Pacing and Electrophysiology. Eur. Heart J. 17, 354–381 10.1093/oxfordjournals.eurheartj.a014868 8737210

[B42] Thayer J. F. Yamamoto S. S. Brosschot J. F. (2010). The relationship of autonomic imbalance, heart rate variability and cardiovascular disease risk factors. Int. J. Cardiol. 141, 122–131 10.1016/j.ijcard.2009.09.543 19910061

[B43] Thiyagarajan R. Pal P. Pal G. K. Subramanian S. K. Bobby Z. Das A. K. (2013). Cardiovagal modulation, oxidative stress, and cardiovascular risk factors in prehypertensive subjects: cross-sectional study. Am. J. Hypertens. 26, 850–857 10.1093/ajh/hpt025 23466463

[B44] Tompkins W. J. (1993). Biomedical Digital Signal Processing: C-Language Examples and Laboratory Experiments for the IBM PC. Englewood Cliffs, NJ: Prentice Hall

[B45] Tsuji H. Larson M. G. Venditti F. J. Manders E. S. Evans J. C. Feldman C. L. (1996). Impact of reduced heart rate variability on risk for cardiac Events: The Framingham Heart Study. Circulation 94, 2850–2855 10.1161/01.CIR.94.11.2850 8941112

[B46] Tulppo M. P. Mäkikallio T. H. Seppänen T. Airaksinen J. K. E. Huikuri H. V. (1998). Heart rate dynamics during accentuated sympathovagal interaction. Am. J. Physiol. Heart Circ. Physiol. 274, H810–H816 9530192 10.1152/ajpheart.1998.274.3.H810

[B47] Tulppo M. P. Mäkikallio T. H. Takala T. E. S. Seppänen T. (1996). Quantitative beat-to-beat analysis of heart rate dynamics during exercise. Am. J. Physiol. Heart Circ. Physiol. 271, H244–H252 8760181 10.1152/ajpheart.1996.271.1.H244

[B48] Voss A. Schulz S. Schroeder R. Baumert M. Caminal P. (2009). Methods derived from nonlinear dynamics for analysing heart rate variability. Philos. Trans. R. Soc. A Math. Phys. Eng. Sci. 367, 277–296 10.1098/rsta.2008.0232 18977726

[B49] Welch P. D. (1967). The use of Fast Fourier Transform for the esimation of power spectra: a method based on time averaging over short, modified periodograms. IEEE Trans. Audio Electroacoust. 15, 70–73 10.1109/TAU.1967.1161901

[B50] Wilson P. W. F. D'Agostino R. B. Levy D. Belanger A. M. Silbershatz H. Kannel W. B. (1998). Prediction of coronary heart disease using risk factor categories. Circulation 97, 1837–1847 9603539 10.1161/01.cir.97.18.1837

[B51] Yoo C. S. Lee K. Yi S. H. Kim J. S. Kim H. C. (2011). Association of heart rate variability with the Framingham risk score in healthy adults. Korean J. Fam. Med. 32, 334–340 10.4082/kjfm.2011.32.6.334 22745871 PMC3383143

